# Complete genome sequence of *Algimonas porphyrae* type strain LMG 26424, a prosthecate bacterium isolated from the red alga *Porphyra yezoensis*

**DOI:** 10.1128/mra.00985-24

**Published:** 2024-10-21

**Authors:** Breah LaSarre, Eva M. Kierstead, Emma M. Corby, Amelia M. Randich

**Affiliations:** 1 Department of Plant Pathology, Entomology, and Microbiology, Iowa State University, Ames, Iowa, USA; 2 Department of Biology, University of Scranton, Scranton, Pennsylvania, USA; DOE Joint Genome Institute, Berkeley, California, USA

**Keywords:** Alphaproteobacteria, Caulobacterales, *Robigintomaculaceae*, prosthecate, alga-associated

## Abstract

*Algimonas porphyrae* is a dimorphic, prosthecate member of the family *Robigintomaculaceae*, order Caulobacterales, in the class Alphaproteobacteria, originally isolated from the red alga *Porphyra yezoensis*. Here we report the complete genome of type strain LMG 26424^T^ (0 C-2-2^T^) obtained by sequencing with Oxford Nanopore technology.

## ANNOUNCEMENT

Caulobacterales order members differ in the location, function, and number of prostheca, which are narrow extensions of the entire cell envelope. *Algimonas porphyrae* is a prosthecate, dimorphic bacterium that was isolated from the red alga *Porphyra yezoensis* in 2013 and originally assigned to the family *Hyphomonadaceae* (1). The *Algimonas* genus is now assigned to *Robigintomaculaceae*, along with the genera *Robiginitomaculum*, *Hellea*, and *Litorimonas* (2). This family exhibits noteworthy variation in reproductive strategies: *Algimonas* is reported to divide by binary fission while its closest relatives by 16S rRNA similarity, *Litorimonas taeanensis* G5T (96.5%) and *Hellea balneolensis* 26III/A02/215T (94.3%) (1), are reported to divide by budding (3, 4). While draft genomes exist for several *Robigintomaculaceae* members, no complete genome has been assembled for any genus of this family. The complete genome sequence of *A. porphyrae* will support efforts to understand variation in the prosthecate morphology and reproductive strategies in this family.

Strain LMG 26424 was grown from a single colony from a DMSO stock (propagated from lyophilisate from BCCM/LMG) in Marine Broth (BD) at 21.5°C, 220 rpm, until OD_600_ 1.34. Cells were washed in phosphate-buffered saline (PBS), resuspended in DNA/RNA Shield (Zymo Research), and sent to Plasmidsaurus for DNA isolation, library preparation, and sequencing using Quick-DNA Miniprep Plus Kit (Zymo), Oxford Nanopore Technologies (ONT) Rapid Barcoding Kit 96 V14, and PromethION P24 with R10.4.1. flow cell, respectively. Super-accurate basecalling (ont-doradod-for-promethion v7.1.4; model dna_r10.4.1_e8.2_400bps_sup@v4.2.0) with read Q-score cutoff of 10 yielded 22,632 raw reads (*N_50_*: 17,592 bp). Reads were filtered using Filtlong v0.2.1 (https://github.com/rrwick/Filtlong) to keep the best 95% of reads >1,000 bp (--keep_percent 95 min_length 1000). The 14,453 filtered reads (*N_50_*: 18,490 bp) were independently assembled using Flye v2.9.3 (5), Raven v1.8.3 (6), Miniasm +Minipolish v0.3-r179/0.1.3 (7, 8), and Canu v2.2 (9). The resulting four assemblies were incorporated into a consensus genome using Trycycler v0.5.4 (10) following the online documentation (https://github.com/rrwick/Trycycler/wiki). Following polishing using Medaka v1.11.3 (https://github.com/nanoporetech/medaka; ONT), the single contig was manually rotated to start at the *dnaA* gene as predicted by bakta v1.9.3 (11). Default parameters were used for all programs unless otherwise noted.

The final assembly comprises a single, circular 3,169,281 bp chromosome (59.31% GC), with estimated 99.84% completeness and 0.16% contamination [per CheckM2 v1.0.2 (12)], and average read coverage of 59× [per Quast v5.2.0 (13)]. Plasmid DNA extraction with a Zyppy Plasmid Kit (Zymo) confirmed the absence of plasmids in LMG 26424. Genes were annotated using the NCBI Prokaryotic Genome Annotation Pipeline during assembly submission to Genbank, with 3,007 protein-coding genes predicted (Table 1). These data are in general agreement with the previous *A. porphyrae* draft genome (Table 1).

**TABLE 1:** Genomic features of the previous draft genome assembly and new complete genome assembly of *A. porpyhyrae* LMG 26424 from RefSeq assembly data

	*A. porphyrae* LMG 26424^*a*^ (this work)	*A. porphyrae* NBRC 108216^*a*^ (previous draft)
Assembly accession No.	GCF_041429795.1	GCF_030159935.1
Assembly level (number of scaffolds)	Complete (1)	Scaffold (55)
Genome size (bp)	3,169,281	3,168,678
% GC	59.31	59.29
Total genes	3,079	3,097
Protein	3,007	3,027
rRNA	3	4
tRNA	41	41
Other RNA	3	3
Total pseudogenes	25	22

^*a*^ LMG 26424 and NBRC 198216 are synonymous strain numbers for type strain 0 C-2-2T.

## Data Availability

This assembled genome was deposited at NCBI’s genome portal under assembly no. CP163424. Raw data are available at NCBI’s SRA portal under BioProject accession no. PRJNA1138649, BioSample accession no. SAMN42730190, and SRA accession number SRS22085969.
